# A Vehicle‐Free Antimicrobial Polymer Hybrid Gold Nanoparticle as Synergistically Therapeutic Platforms for *Staphylococcus aureus* Infected Wound Healing

**DOI:** 10.1002/advs.202105223

**Published:** 2022-03-10

**Authors:** Xiaojun He, Lixiong Dai, Lisong Ye, Xiaoshuai Sun, Obeng Enoch, Rongdang Hu, Xingjie Zan, Feng Lin, Jianliang Shen

**Affiliations:** ^1^ School of Ophthalmology & Optometry School of Biomedical Engineering Wenzhou Medical University Wenzhou Zhejiang 325035 China; ^2^ Wenzhou Institute University of Chinese Academy of Sciences Wenzhou 325000 China; ^3^ School of Stomatology Wenzhou Medical University Wenzhou Zhejiang 325035 China; ^4^ Oujiang Laboratory (Zhejiang Lab for Regenerative Medicine, Vision and Brain Health) Wenzhou Zhejiang 325001 China; ^5^ Department of gynecology The First Affiliated Hospital of Wenzhou Medical University Wenzhou 325000 China

**Keywords:** anti‐biofilm, bactericidal effect, hybrid gold nanoparticles, polyhexamethylene biguanide, wound healing

## Abstract

Pathogenic bacteria infection is a serious threat to human public health due to the high morbidity and mortality rates. Nano delivery system for delivering antibiotics provides an alternative option to improve the efficiency compared to conventional therapeutic agents. In addition to the drug loading capacity of nanocarriers, which is typically around 10%, further lowers the drug dose that pathological bacteria are exposed to. Moreover, nanocarriers that are not eliminated from the body may cause side effects. These limitations have motivated the development of self‐delivery systems that are formed by the self‐assembly of different therapeutic agents. In this study, a vehicle‐free antimicrobial polymer polyhexamethylene biguanide (PHMB, with bactericidal and anti‐biofilm functions) hybrid gold nanoparticle (Au NPs, with photothermal therapy (PTT)) platform (PHMB@Au NPs) is developed. This platform exhibits an excellent synergistic effect to enhance the photothermal bactericidal effect for *Staphylococcus aureus* under near‐infrared irradiation. Furthermore, the results showed that PHMB@Au NPs inhibit the formation of biofilms, quickly remove bacteria to promote wound healing through PTT in infection model in vivo, and even mediate the transition of macrophages from M1 to M2 type, and accelerate tissue angiogenesis. PHMB@Au NPs will have promising value as highly effective antimicrobial agents for patient management.

## Graphical abstract

A vehicle‐free antimicrobial polymer polyhexamethylene biguanide (PHMB, with bactericidal and anti‐biofilm functions) hybrid gold nanoparticle (Au NPs, with photothermal therapy) platform (PHMB@Au NPs) exhibits synergistic interaction and enhanced photothermal bactericidal effect and anti‐biofilm for *Staphylococcus aureus* under near‐infrared irradiation with superior biosecurity.

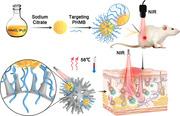

## 1 Introduction

*Staphylococcus aureus* (*S. aureus*) acts as a common conditional pathogen. The infection of this pathogen can cause a series of diseases, and its severe drug resistance brings great difficulties to clinical treatment.^[^ ^1^ ^]^ The long‐term or large‐scale use of antibiotics causes the bacteria to develop resistance, leading to the emergence of a large number of resistant bacteria. the “super bacteria”, such as *Methicillin‐Resistant S. aureus* (MRSA) have made the selection of antibiotics more and more advanced, and the dosage has increased, which has increased the burden on patients, and the side effects of drugs have also increased.^[^ ^2^ ^]^ Therefore, facing the increasingly serious problem of bacterial resistance, it is urgent to seek new broad‐spectrum, high‐efficiency, and low‐toxicity anti‐drug‐resistant bacteria drugs and antimicrobial strategies.^[^ ^3^ ^]^ The method of delivering antibiotics or antibacterial drugs through nanomaterials to reduce the dose of drugs while increasing the antibacterial efficiency is considered to be a promising antibacterial strategy.^[^ ^4^ ^]^ At present, most nanocarriers such as Si, SiO_2_, ZrO_2_, and TiO_2_ nanomaterials are widely used as drug carriers.^[^ ^5^ ^]^ Due to the low load of these carriers, the biodistribution is complicated, and it is difficult to avoid the side effects of the carriers themselves. These bottlenecks have actively promoted the development of self‐delivery systems.

However, among the nanomaterials of photothermal therapy (PTT), gold nanoparticles (Au NPs) have received extensive attention in the field of photothermal antitumor and antimicrobial applications due to their satisfactory photothermal performance and excellent biosecurity.^[^ ^6^ ^]^ Compared with other nanoparticles, Au NPs are nanomaterials whose surface is easy to be directly modified, which greatly promotes the development of new antimicrobial material.^[^ ^7^ ^]^ Free Au NPs do not have the function of antibacterial activity, but the biological activity of Au NPs can be improved by photothermal or functional modification.^[^ ^8^ ^]^ Moreover, Au NPs of small size or surface charge are not stable enough, they are easy to accumulate and lead to a sharp drop in photothermal performance. Therefore, most researchers improve the photothermal performance and biological activity of nanomaterials by modifying the surface activity of Au NPs and small molecules.^[^ ^9^ ^]^ Among them, polyhexamethylene biguanide (PHMB) was widely used in medical products, for example disinfecting eye drops, contact lenses, and disinfectants.^[^ ^10^ ^]^ According to previous research reports, PHMB can destroy the cell membrane structure of bacteria by non‐specifically binding fatty acids on the cell membrane.^[^ ^11^ ^]^ Additionally, PHMB can cause cell wall damage, reduced membrane fluidity, ions, ATP, and protein leak from the cell, eventually leading to the death of bacteria. Nevertheless, it is difficult to avoid concentration dependence during clinical therapeutics, and excessive use of PHMB will bring certain side effects.

**Scheme 1:**
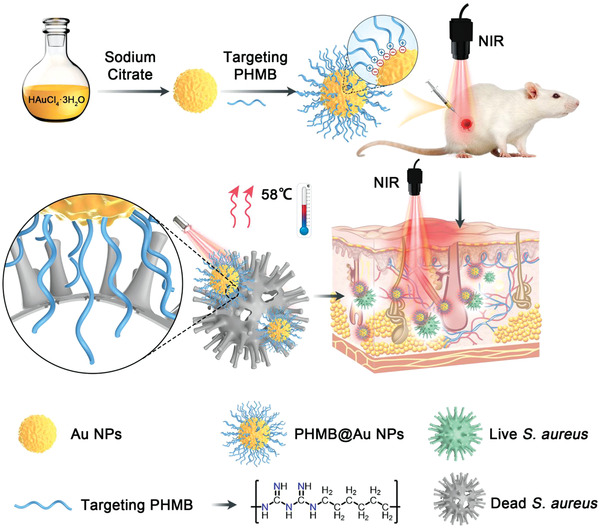
Schematic illustration of Au NPs and PHMB@Au NPs fabrication, and evaluation of the mechanism of bactericidal effect in vivo to promote healing of infected wounds.

Herein, to develop new high‐efficiency antibacterial agents and antibacterial strategies, the self‐assembly of PHMB and Au NPs through a vehicle‐free strategy was achieved self‐delivery. The self‐delivery system was confirmed by characterization, and the antibacterial and anti‐biofilm activities of PHMB@Au NPs against *S. aureus* were studied. The results showed that the functionalized PHMB@Au NPs can quickly kill *S. aureus* and destroy the formation of biofilm. Through the subcutaneous abscess and wound healing model, it is strongly proved that PHMB@Au NPs can remove bacteria in the subcutaneous tissue under irradiation. Meanwhile, immunofluorescence analysis found that PHMB@Au NPs can mediate the conversion of M1 macrophages to M2 macrophages, and promote angiogenesis to accelerate the rapid healing of infected wounds (**Scheme** **1**). This work will promote the development of Au NPs in the field of self‐delivery systems.

## 2 Results and Discussion

**Figure 1:**
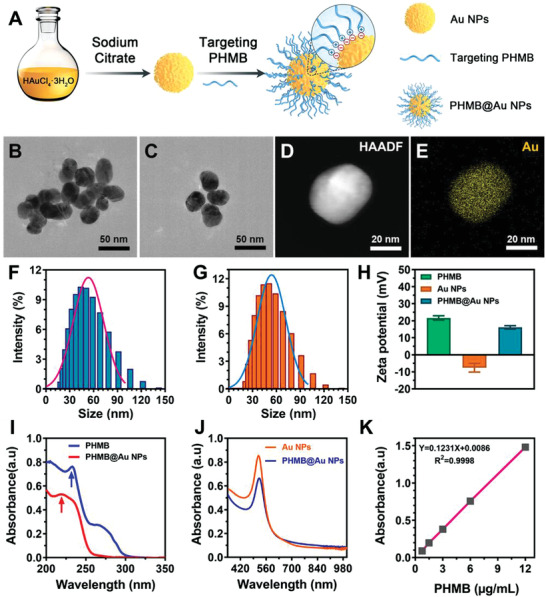
Characterization of PHMB‐derivative‐modified Au NPs. A) Schematic illustration of PHMB@Au NPs fabrication. TEM images of the B) Au NPs and C) with PHMB modifications. Scale bars = 50 nm. D) STEM image of PHMB@Au NPs and E) elemental mappings of Au. Scale bars = 20 nm. F) DLS analysis of the Au NPs and G) modified by PHMB. H) Zeta potential of Au NPs and PHMB@Au NPs. I,J) UV–vis spectrum of PHMB, Au NPs, and PHMB@Au NPs. K) The calibration curve of the concentration of PHMB.

Au NPs have superior photothermal properties and good biocompatibility, which are widely used as a building block for various biological materials.^[^ ^12^ ^]^ Therefore, Au NPs can form stable nano‐systems through self‐assembly and functionalized polymers, realizing the zero‐carrier strategy of drug‐loading drugs. This shows that not only improves the dispersibility of Au NPs but also endow Au NPs with better biological activity. Features, PHMB is a positively charged polymer, which can effectively target the membrane of bacteria and has high‐efficiency broad‐spectrum antimicrobial activity. Therefore, PHMB combines with the negatively charged Au NPs through electrostatic interaction to self‐assembly into functionally modified PHMB@Au NPs. **Figure** **1**A illustrates the synthesis process of PHMB@Au NPs and the self‐assembling driving force of the electrostatic interaction mode of the two. PHMB@Au NPs are prepared according to the method previously reported.^[^ ^6^ ^]^ First, we use citric acid to reduce AuCl_4_ into spherical Au NPs with a diameter of 50 nm. Then PHMB is used to bind with Au NPs, and the potential synthesis mechanism of the coupling is electrostatic attraction. Moreover, the self‐assembly of PHMB with Au NPs at a relatively low concentration resulted in a PHMB@Au NPs system with well antibacterial activity, indicating that the effective antibacterial concentration of PHMB can be reduced through synergistic action, thereby having the potential to reduce side effects.^[^ ^13^ ^]^ Transmission electron microscopy (TEM) images revealed that both Au NPs and PHMB@Au NPs are spherical and have a diameter of about 50 nm (Figure 1B,C). In addition, the element distribution of PHMB@Au NPs was characterized by TEM combined with high‐resolution transmission electron microscopy (HRTEM) images. As shown in the elemental mapping images (Figure 1D,E), the distribution of Au further indicates that Au is the main nanoparticle carrier. It can also be observed that some of the C and N elements are enriched in Au NPs (Figure S1, Supporting Information). After functionalization with PHMB, Au NPs maintained almost the same shape and size. The change of the particle size before and after modification of the Au NPs by PHMB in the solution was monitored by dynamic light scattering. Figure 1F,G shows that the PHMB‐modified Au NPs are spherical with good dispersion and no obvious aggregation. The obtained PHMB@Au NPs were transparent and homogeneous in phosphate‐buffered saline (PBS) without precipitation. To investigate the surface modification process, the spectral characteristics and surface charge of PHMB@Au NPs were confirmed by zeta potential analysis and ultraviolet‐visible‐near infrared (UV–vis‐NIR) spectroscopy. Due to the presence of residual citrate molecules, the zeta potentials of PHMB and Au NPs are +22.0 and −9.8 mV, respectively (Figure 1H). After PHMB was functionalized, the zeta potential increased to +15.0 mV, indicating that the citrate molecule was replaced by PHMB. Au NPs and PHMB and Au NPs have a peak at 230 and 520 nm in the UV–vis‐NIR spectrum (Figure 1I,J), which correspond to the characteristic peaks of PHMB and Au NPs, respectively. The characteristic absorption peaks of PHMB and PHMB@Au NP are somewhat shifted, which is due to the increase of the local refractive index of the modified with PHMB on Au NPs, resulting in the red or blue shift. Hence, we quantified the content of PHMB by detecting the absorption at 230 nm and further determined that the PHMB content of PHMB@Au NPs loaded with PHMB was 4.3 µg mL^−1^ through the standard curve of PHMB (Figure 1K). Furthermore, we can accurately calculate that the PHMB contained in PHMB@Au NPs was 4.3 µg mL^−1^, which has no antibacterial activity with PHMB alone under this concentration (Figure S2, Supporting Information).

**Figure 2:**
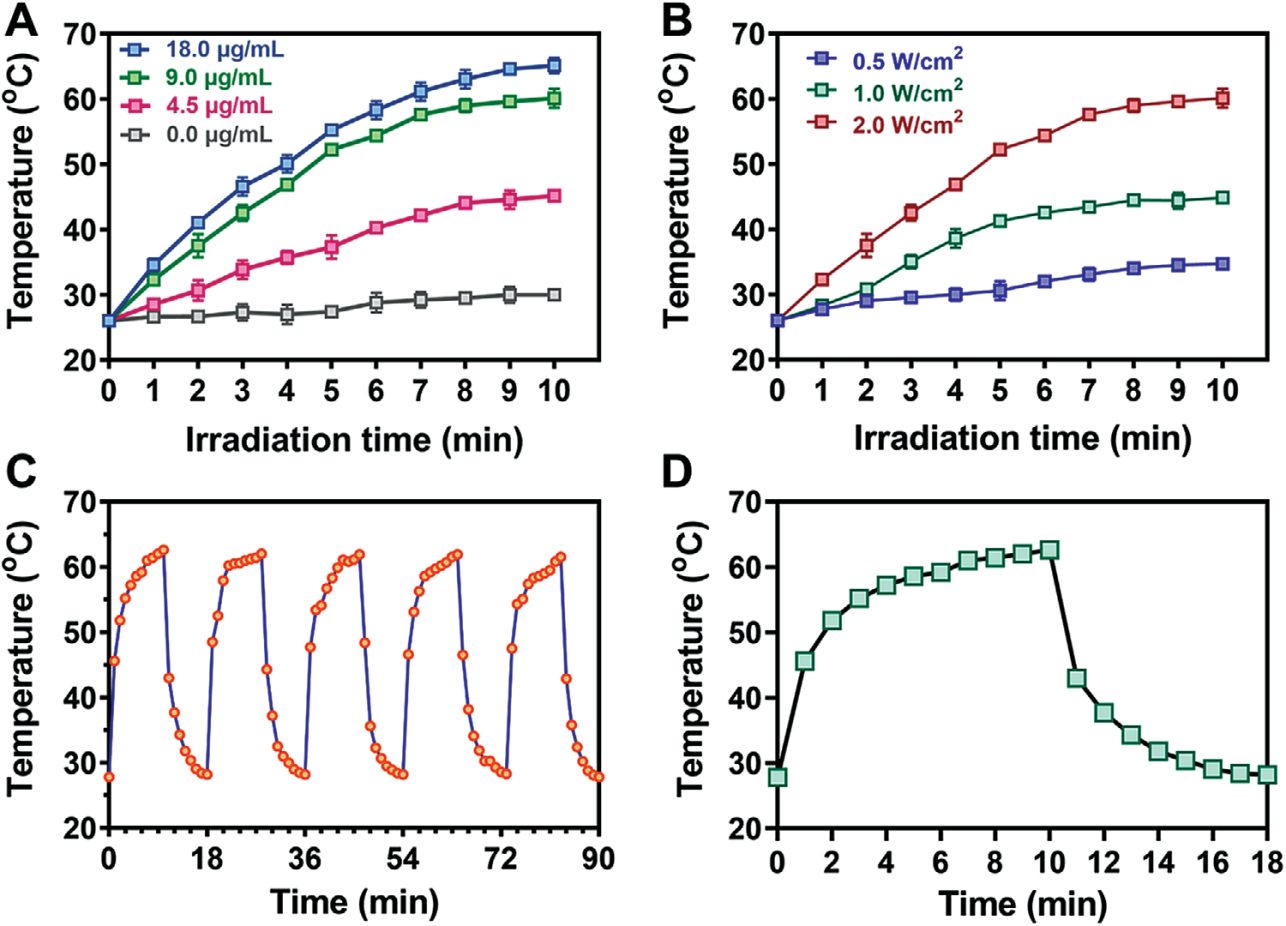
Photothermal performance of PHMB@Au NPs. A) Photothermal heating curves of PHMB@Au NPs at different concentrations under NIR irradiation (808 nm laser, 2.0 W cm^−2^). B) Temperature evolution curves of PHMB@Au NPs (9.0 µg mL^−1^) solutions under the different intensities of NIR irradiation. C) Heating and cooling cycles of PHMB@Au NPs. D) The temperature of the PHMB@Au NPs dispersions rose until reaching a stable temperature and then cooled down naturally.

Au‐based materials have been previously reported as efficient photothermal conversion intermediaries. Consequently, the photothermal performance of functionalized Au‐based nanoparticles was roundly assessed. The photothermal performance is studied by monitoring the variation of water temperature under the NIR irradiation of a laser with 808 nm (2.0 W cm^−2^) for 10 min. As shown in **Figure** **2**A, PHMB@Au NPs show concentration‐dependent temperature changes under NIR radiation (2.0 W cm^−2^). Specifically, the temperature increased rapidly from 26.0 to 45.0 °C (4.5 µg mL^−1^), 58.4 °C (9.0 µg mL^−1^), 65.2 °C (18.0 µg mL^−1^), and for free PBS, the temperature increased only slightly to 30.2 °C. Corresponding thermal images can visually present temperature changes (Figures S3 and S4, Supporting Information). The results show that the photothermal conversion efficiency of PHMB@Au NPs is concentration‐dependent. Moreover, PHMB@Au NPs (9.0 µg mL^−1^) showed a gradient in temperature changes under the irradiation of 808 nm lasers with different powers. It shows that the temperature change of PHMB@Au NPs exhibits photothermal performance related to power density (Figure 2B). Corresponding thermal images can visually present temperature changes (Figure S5, Supporting Information). The photothermal performance of PHMB@Au NPs shows insignificant degradation during the five lasers on/off cycles (Figure 2C), highlighting the well photothermal performance of PHMB@Au NPs in potential continuous photothermal elimination of bacteria. According to the heating and cooling curve (Figure 2D) and the corresponding thermal time constant (*τ*_s_), the photothermal conversion efficiency (*η*) is determined to be 54.2% according to formulas (1), (2), and (3). The above results show that the functionalized PHMB@Au NPs have good photothermal performance and basic conditions for photothermal antimicrobial activity.

Before the antimicrobial activity experiment, the concentration of PHMB@Au NPs and laser parameters were determined by phototoxicity testing on mammalian cells. The cell viability of PHMB@Au NPs on fibroblasts cells (L929) was measured using 5‐diphenyl‐2‐H‐tetrazolium bromide (MTT) assay. Before checking cell viability, incubate L929 cells with different concentrations of PHMB@Au NP under NIR irradiation (2.0 W cm^−2^) for 10 min. Even if the concentration of PHMB@Au NPs is as high as 18.0 µg mL^−1^ (Figure S6A, Supporting Information), the cell viability is still higher than 90% of the control, which indicates the cytotoxicity of the sample is low. A laser power of 0 to 2.0 W cm^−2^ was used to evaluate the phototoxicity of PHMB@Au NPs (18.0 µg mL^−1^) (Figure S6B, Supporting Information). There is no doubt that low toxicity is essential for PTT in biomedical applications. In the following antimicrobial measurement, unless otherwise specified, the concentration is set to 9.0 µg mL^−1^, the laser power is set to 2.0 W cm^−2^, and the duration is 5 min.

**Figure 3:**
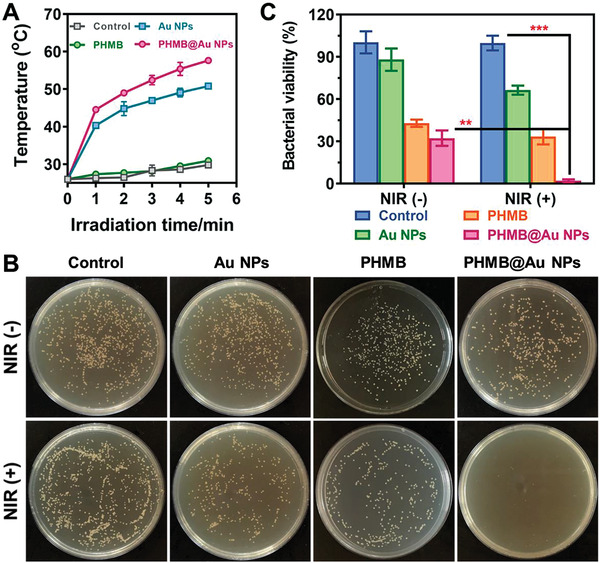
Antimicrobial activity of PHMB@Au NPs in vitro. A) Temperature changes of *S. aureus* solution during treatment by PBS, Au NPs, PHMB, and PHMB@Au NPs at the concentration of 9.0 µg mL^−1^ under 808 nm laser irradiation (2.0 W cm^−2^). B) Photographic images of the colonies of *S. aureus* treated by different methods on TSB‐agar plates. The laser power density was 2.0 W cm^−2^, without and with exposure for 5 min of 808 nm laser. C) Bacterial viability of *S. aureus* treated by different methods (n = 3). **P <0.01 and ***P <0.001.

To evaluate the antimicrobial activity of PHMB@Au NPs against the typical pathogen *S. aureus* in vitro, the plate counting method was used. The PHMB, Au NPs, and PHMB@Au NPs were incubated with *S. aureus* and irradiated with an 808 nm laser. As shown in **Figure** **3**A, after the Au NPs and PHMB@Au NPs are treated with the *S. aureus*, the system temperature is significantly higher under laser irradiation, and the height temperature of the PHMB@Au NPs system is higher than that of the Au NPs system, which is because the PHMB modification makes Au NPs have Better dispersibility, and helps bacteria to adhere and swallow. There was almost no significant temperature increase in the control group and the PHMB group. It is worth noting that in the absence of laser irradiation, Au NPs have insignificant antimicrobial activity. However, PHMB and PHMB@Au NPs show certain antimicrobial activity, and their inhibition rate against bacteria is about 70%, mainly from free PHMB (Figure 3B). However, PHMB@Au NPs showed unexpected antimicrobial activity under 808 nm laser irradiation, and its inhibition rate of bacteria was as high as 95% or more (Figure 3C). In this process, PHMB can enhance the adhesion of PHMB@Au NPs to bacteria, and promote the penetration of the bacterial cell wall by physical or chemical strategies of drugs, leading to bacterial leakage. After laser irradiation, the bactericidal activity of PHMB@Au NPs is significantly enhanced due to the effect of PTT. In summary, the PHMB functionalized Au NPs specifically target bacteria and exhibit superior antimicrobial activity.

**Figure 4:**
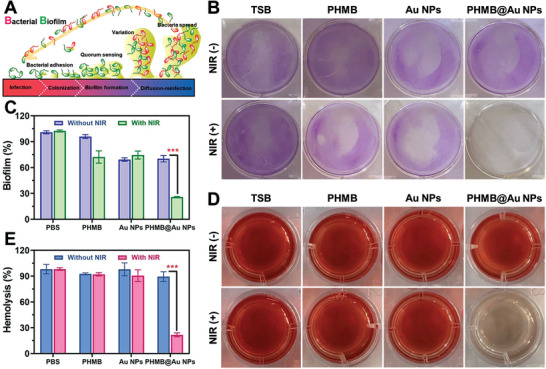
Inhibition of biofilm and hemolysis of *S. aureus*. A) Schematic illustration of biofilm formation process of *S. aureus*. B) Digital images of biofilm formation of *S. aureus* with the addition of TSB, PHMB, Au NPs, and PHMB@Au NPs under NIR (808 nm, 2.0 W cm^−2^) for 24 h. C) Percentage quantitative biofilm formation under different conditions (n = 3). D) Photograph of hemolysin activity of *S. aureus* with the addition of trypticase soy broth (TSB), PHMB, Au NPs, and PHMB@Au NPs under NIR (808 nm, 2.0 W cm^−2^) for 24 h. E) Percentage quantitative hemolysin activity under different conditions (n = 3). ***P <0.001.

The formation of bacterial biofilm is one of the most critical factors leading to drug resistance, and the formation of bacterial biofilm further prevents antimicrobial drugs from contacting bacteria, thereby delaying the healing of infected wounds (**Figure** **4**A). To evaluate the influence of PHMB@Au NPs on the formation of *S. aureus* biofilms, first, the bacteria were treated with different materials and then cultured for 24 h. The adhered biofilms were stained with crystal violet (CV) and quantified. As demonstrated in Figure 4B,C, PHMB@Au NPs treated group reduced biofilm formation by approximately 85% under NIR irradiation compared with the control. In contrast, the formation of biofilm under other conditions only slightly decreased. The above results demonstrated that PHMB@Au NPs can effectively inhibit the formation of bacterial biofilms, indicating that the material has the potential to eliminate drug‐resistant bacteria.

*S. aureus* can cause local purulent infections, sepsis, and sepsis in humans and animals, and its main pathogenic factors include hemolysin, including *α*‐ and *β*‐hemolysin. Therefore, judging that an antimicrobial drug can effectively inhibit the toxicity of *S. aureus* is also an important indicator for judging its antibacterial activity. The bacteria were treated with different materials with NIR irradiation. High‐speed centrifugation, the ability of bacterial hemolysin in the supernatant medium to lyse rabbit red blood cells was observed. As shown in Figure 4D,E, the hemolysin secreted by *S. aureus* in the PHMB@Au NPs treatment group was significantly inhibited after NIR irradiation, which was reduced by more than 80% compared to the control group. On the contrary, the other condition groups had almost no inhibitory effect on the secretion of bacterial hemolysin. All these results indicate that PHMB@Au NPs have a strong inhibitory effect on the secretion of *S. aureus* hemolysin.

**Figure 5:**
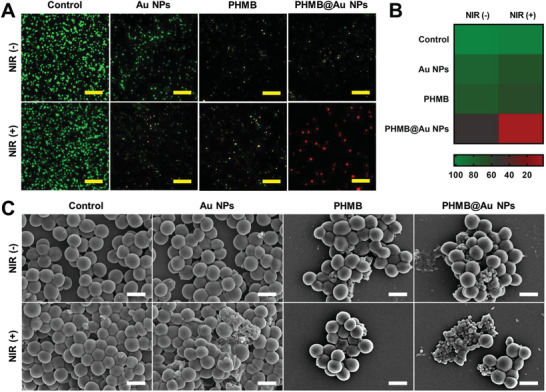
Morphological change of *S. aureus* after incubated with PHMB@Au NPs. A) Living/dead fluorescence images of *S. aureus* after being treated by different methods (Living/dead cells are stained with green/red fluorescence respectively, scale bar: 20 µm). B) Quantized schematic illustration of the fluorescence intensity ratio (Fgreen/Fred) under different materials without and with NIR irradiation for living/dead assay. C) SEM images of *S. aureus* after 5 min of 808 nm laser irradiation (2.0 W cm^−2^) with the treatment of PHMB@Au NPs (9.0 µg mL^−1^). The control groups were imaged in the absence and presence of irradiation treatment with a TSB medium. scale bar: 1.0 µm.

Insight into exploring the antimicrobial mechanism of PHMB@Au NPs, a live/dead staining test was carried out. In this assay, observed under fluorescence imaging, live bacteria with intact bacterial cell membranes present green fluorescence signals, while dead bacteria with damaged bacterial cell membranes present red fluorescence signals. In the absence of NIR irradiation, the bacteria showed green fluorescence after treatment with various materials, indicating that the bacterial activity was unaffected. The bacteria treated with PHMB and PHMB@Au NPs only produced part of the red fluorescence. However, in the presence of NIR irradiation, PHMB@Au NPs treated *S. aureus* and most of the bacteria showed red fluorescence, indicating an increase in the number of damaged or dead bacteria (**Figure** **5**A), which is consistent with the results of the standard plate count determination. According to reports, the enzymes, proteins, and lipids in the bacteria will be denatured, metabolic disorders will eventually lead to the death of the bacteria above 50 °C.

Furthermore, by quantifying the fluorescence intensity ratio (*F*_green_/*F*_red_) of living/dead assay staining, which was revealed that 90% of the bacteria have died under NIR irradiation for 5 min (Figure 5B). Bacterial live/dead staining test showed that after PHMB@Au NPs were exposed to NIR irradiation, the number of live bacteria was greatly reduced, indicating that PHMB@Au NPs had a photothermal sterilization effect under NIR irradiation. To explore the antimicrobial mechanism of the material from the morphology of bacteria, the integrity of the bacterial cell wall was evaluated by the SEM method. As shown in Figure 5C, in the absence of NIR irradiation and the presence of various materials, the cell walls of bacteria are smooth and spherical (local zoom) (Figure S7, Supporting Information). After PHMB and PHMB@Au NPs are treated, some of the surfaces of bacteria begin to wrinkle or dent, indicating that the bacteria are beginning to die. However, in the presence of NIR irradiation, PHMB@Au NPs treated *S. aureus*, leaving most of the bacterial cell walls in a damaged state, indicating an increase in the number of dead or damaged bacteria. In summary, from the live/dead staining of bacteria and SEM experiments, the antimicrobial mechanism is attributed to the direct electrostatic interaction between the sharp edges of the PHMB@Au NPs and bacteria membrane surfaces, and then through photothermal antimicrobial therapy to make the material have high antimicrobial activity.

**Figure 6:**
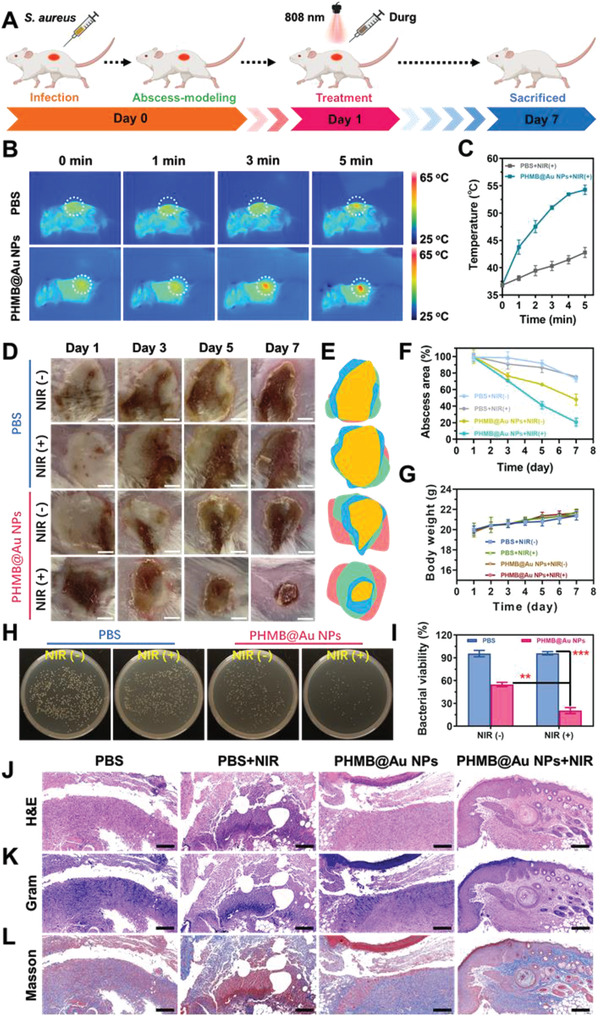
Antimicrobial activity of PHMB@Au NPs in vivo with subcutaneous infection model. A) Schematic diagram of PHMB@Au NPs for the antibacterial therapeutic process in vivo. B) Infrared thermal images and C) corresponding temperature elevation curves of mice after different treatments under NIR irradiation (808 nm, 2.0 W cm^−2^) from 0 to 5 min. D) Thermographic images of mice treated by PBS and PHMB@Au NPs under NIR laser irradiation for 5 min. Scale bar: 200 mm. E) Fractions of the wounds healed by the different treatments on day 1, day 3, day 5, and day 7. F) Corresponding abscess areas of infected mice after treatment with different materials. G) Time‐dependent body‐weight curves of mice after different treatments. H) Photographs of bacterial colonies obtained from infected tissues of mice treated by different methods and I) Quantitative statistics of the number of bacterial colonies through standard plate counting assay (n = 3). **P <0.01 and ***P <0.001. The infected skin histologic analyses, including J) H&E, K) Gram stain, and L) Masson on day 7 with treatments. Scale bar, 200 µm.

To verify antimicrobial activity in vivo, the PHMB@Au NPs system was orthotopic injected into the subcutaneous abscess tissue, which was created in mice model via the infection of *S. aureus* and then irradiated by NIR (808 nm, 2.0 W cm^−2^) as exhibited in **Figure** **6**A. PHMB@Au NPs injected into the skin of *S. aureus*‐infected (experimental group) showed significant temperature increase and remained at about 54 °C under NIR irradiation from 0 to 5 min (Figure 6B,C). This temperature is sufficient for to bactericidal effect, which has been proven in previous studies.^[^ ^14^ ^]^ In addition, the tissue surrounding the bacterial infection did not show a significant temperature increase, which means that there is no thermal damage to the healthy tissue. Without NIR irradiation, mice treated with PBS and PHMB@Au NPs developed severe inflammation and abscesses, indicating that severe local abscesses and ulcers appeared after the bacteria were not killed or eliminated (Figure 6D). Digital photographs of the abscesses area showed that mice treated with PHMB@Au NPs exhibited significantly faster‐wound closure compared to the other groups, with nearly 80% closure achieved on day 7 (Figure 6E,F). The bodyweight of the mice remained stable during treatments and was not recognized to be different between the four groups (Figure 6G). Under the action of NIR irradiation, compared with the PBS group, the inflammation and abscess under the skin were significantly improved after treatment with PHMB@Au NPs for a different time, indicating that the bactericidal effect is good and the wound tissue was healed. Additionally, the bactericidal effect of subcutaneous abscess in each group of mice was then analyzed by using a plate counting assay. After receiving PHMB@Au NPs treatment under NIR irradiation, the colony‐forming unit count showed a praiseworthy bactericidal effect (Figure 6H,I). On the 7th day of wound healing, histological evaluation of granulation tissue formation, bacterial removal, and tissue maturation was performed by measuring the thickness of the granulation tissue, the bacterial content of the wound tissue, and the collagen deposition. Histological analysis showed that the thickness of granulation tissue in the PHMB@Au NPs treatment group was significantly increased compared with other groups (Figure 6J). Moreover, the number of bacteria in the wound tissue gradually decreased with the treatment of drugs (Figure 6K). A large amount of collagen deposition was observed in PHMB@Au NPs‐treated wounds, indicating the recovery and maturation of damaged tissues (Figure 6L).

**Figure 7:**
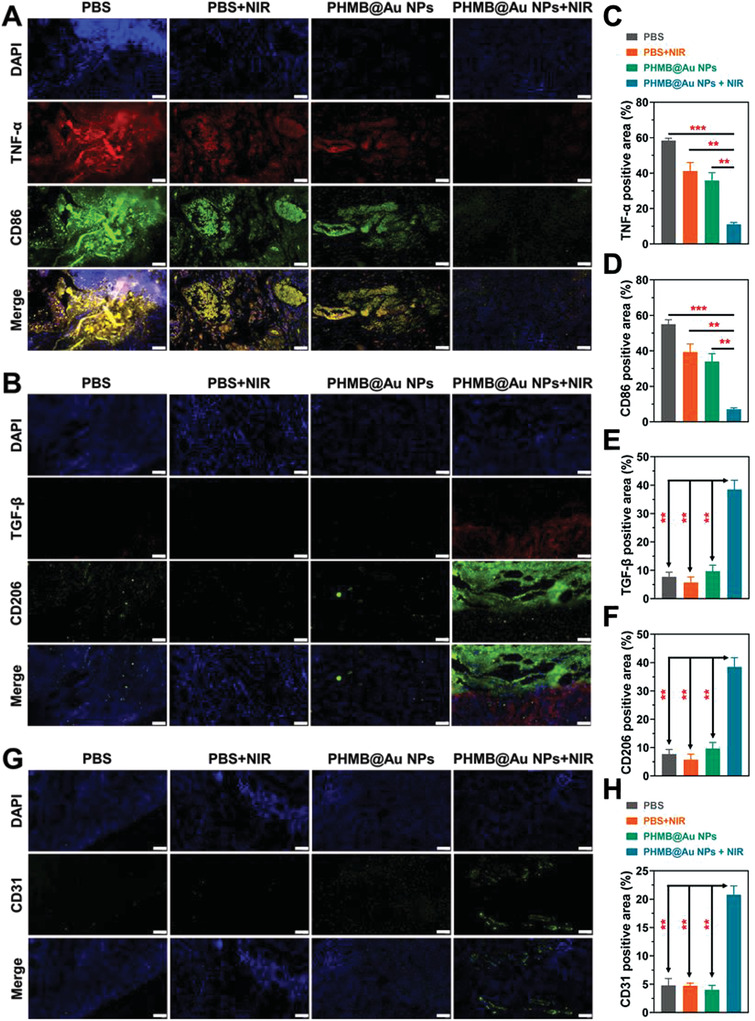
Immunofluorescence analysis of wounds recovery process. Representative images of A) M1 macrophages (TNF‐*α* and CD86) and B) M2 macrophages (TGF‐*β* and CD206) immunofluorescence staining on day 7. Scale bar, 50 µm. C–F) Statistical data of the percentage of TNF‐*α*, CD86, TGF‐*β*, and CD206 (n = 3). G) Representative images of angiogenesis (CD31) immunofluorescence on day 7. Scale bar, 50 µm. H) Statistical data of CD31 positive area in the wound tissue (n = 3). **P <0.01 and ***P <0.001.

The wound healing process includes several stages such as antibacterial, anti‐inflammatory, and promoting angiogenesis. In the process of bacterial infection, certain inflammation will inevitably be caused. Therefore, the inflammatory changes in the wounds of mice during the antibacterial process were explored by immunofluorescence staining of infected tissues. There are two types of macrophages: pro‐inflammatory (M1) and anti‐inflammatory (M2) macrophages, and the markers of the two cells will change to a certain extent during the wound healing process. Therefore, the inflammation of the wound can be observed indirectly by detecting the corresponding markers in the tissue. Therefore, we performed immunofluorescence staining of macrophages in wound sections during the proliferation phase (day 7) to clarify the in vivo role of PHMB@Au NPs in inducing M1 macrophages to the M2 phenotype. As shown in **Figure** **7**A,B, the administration of PHMB@Au NPs significantly reduced the proportion of TNF‐*α* and CD86 M1 labeled positive cells, while compared with other groups, the infiltration and distribution of TGF‐*β* and CD206 M2 labeled positive cells was enhanced up (Figure 7C–F). During wound healing, M2 macrophages secrete anti‐inflammatory mediators and release angiogenesis (marker CD31) as shown in Figure 7G,H. Our results further confirm that PHMB@Au NPs can drive the phenotype of macrophages for tissue healing and regeneration.

**Figure 8:**
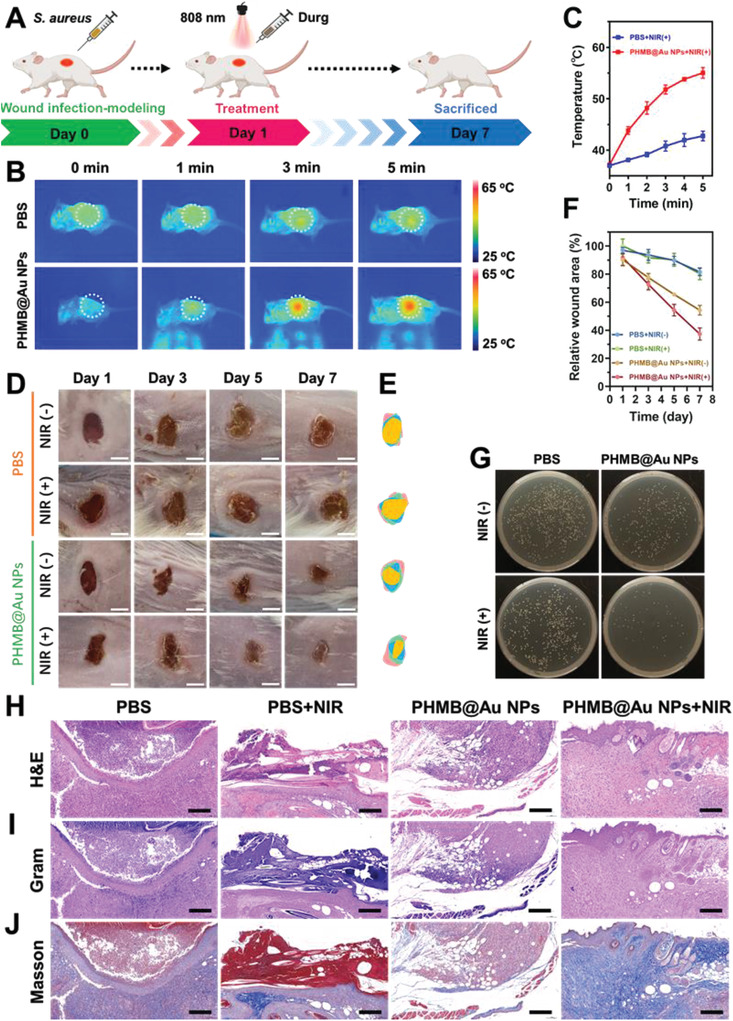
Antimicrobial activity of PHMB@Au NPs in vivo with wound‐healing model. A) Schematic diagram of PHMB@Au NPs for the antibacterial therapeutic process in vivo. B) Infrared thermal images and C) corresponding temperature elevation curves of mice after different treatments under NIR irradiation (808 nm, 2.0 W cm^−2^) from 0 to 5 min. D) Thermographic images of mice treated by PBS and PHMB@Au NPs under NIR laser irradiation for 5 min. Scale bar: 500 mm. E) Fractions of the wounds healed by the different treatments on day 1, day 3, day 5, and day 7. F) Corresponding wound areas of infected mice after treatment with different materials. G) Photographs of bacterial colonies obtained from infected tissues of mice treated by different methods. The infected skin histologic analyses, including H) H&E, I) Gram stain, and J) Masson on day 7 with treatments. Scale bar, 200 µm.

To further confirm the antimicrobial activity in vivo, a wound healing model was established, and PHMB@Au NPs injection combined with NIR irradiation was used (**Figure** **8**A). Mice were anesthetized and spread on a wound with *S. aureus* on their backs. During the photothermal antimicrobial therapy processing, a thermal imager was used to record thermal imaging photos. The photograph was used to evaluate the imaging characteristics of PHMB@Au NPs. The subcutaneous abscesses injected with PHMB@Au NPs showed prominent temperature increased and keep at around 55 °C under NIR irradiation (Figure 8B,C). On the contrary, the tissue surrounding the wound did not show a significant temperature increase, which means that the healthy tissue was not damaged by thermal. Digital photographs of the wound area showed that mice treated with PHMB@Au NPs exhibited significantly faster‐wound closure compared to the other groups (Figures 8D), with nearly 60% closure achieved on day 7 (Figure 8E,F). The bodyweight of the mice remained stable during treatments and was not recognized to be different between the four groups (Figure S8A, Supporting Information). There is no distinct inflammation on the epidermis and dermis, indicating that PHMB@Au NPs have a superior bactericidal effect under NIR irradiation. Inversely, mice in other groups showed severe inflammation and abscesses on their wounds, indicating poor antimicrobial activity makes wounds difficult to heal. The plate counting assay was used to quantitatively evaluate the bactericidal effects of different groups (Figure 8G and Figure S8B, Supporting Information). The examination of the bacterial concentration in the tissue fluid confirmed that PHMB@Au NPs have a superior bactericidal effect under NIR irradiation. The degree of wound healing is analyzed by histology, and granulation tissue formation, bacterial removal, and tissue maturation are evaluated histologically by measuring the thickness of granulation tissue, the bacterial content, and collagen deposition of the wound tissue. Histological analysis showed that the thickness of granulation tissue in the PHMB@Au NPs treatment group was significantly increased compared with other groups (Figure 8H), and there was an obvious formation of hair follicle structure. Moreover, the number of bacteria in the Gram staining statistics of the wound tissue gradually decreased with the treatment of drugs Figure 8I). A large amount of collagen deposition was observed in PHMB@Au NPs‐treated wounds, indicating the recovery and maturation of damaged tissues (Figure 8J). The results show that PHMB@Au NPs under NIR irradiation can effectively kill bacteria and promote wound healing, but no damage to surrounding healthy tissues.

**Figure 9:**
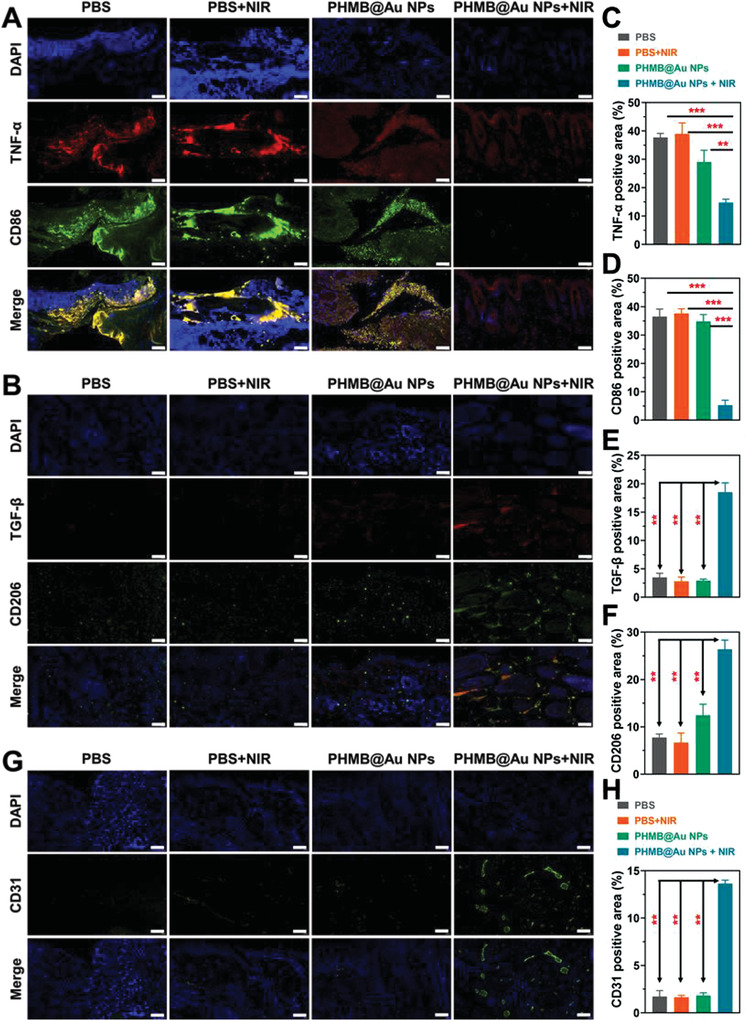
Immunofluorescence analysis of wounds recovery process. Representative images of A) M1 macrophages (TNF‐*α* and CD86) and B) M2 macrophages (TGF‐*β* and CD206) immunofluorescence staining on day 7. Scale bar, 50 µm. C–F) Statistical data of the percentage of TNF‐*α*, CD86, TGF‐*β*, and CD206 (n = 3). G) Representative images of angiogenesis (CD31) immunofluorescence on day 7. Scale bar, 50 µm. H) Statistical data of CD31 positive area in the wound tissue (n = 3). **P <0.01 and ***P <0.001.

Additionally, the markers of M1 macrophages and M2 macrophages are detected by immunofluorescence to feedback the healing process of the wound. In the proliferation stage on the 7th day, the macrophages in the wound section were analyzed by immunofluorescence staining. As shown in **Figure** **9**A,B, the administration of PHMB@Au NPs significantly reduced the TNF‐*α* and CD86 M1 labeled positive cells. Compared with other groups, the infiltration and distribution of TGF‐*β* and CD206 M2 labeled positive cells were enhanced (Figure 9C–F). In the process of wound healing, M2 macrophages secrete anti‐inflammatory mediators and release angiogenesis (marker CD31), as shown in Figure 9G,H, indicating that the wound gradually begins to heal after antibacterial treatment. The above results indicate that PHMB@Au NPs can drive the phenotype of macrophages for tissue healing and regeneration.

**Figure 10:**
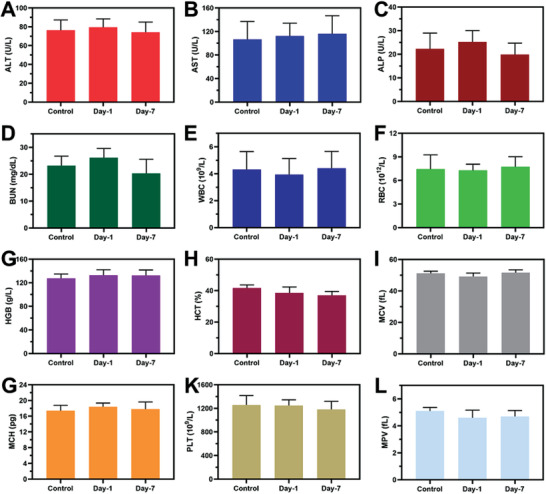
Biosafety evaluations of PHMB@Au NPs in Vivo. Blood biochemistry examination, including A) ALT, B) AST, C) ALP, and D) BUN. Blood routine examination, including E) WBC, F) RBC, G) HGB, H) HCT, I) MCV, G) MCH, K) PLT, and L) MPV of healthy mice after subcutaneous injection under different treatment conditions at day‐0, day‐1, and day‐7.

Biosafety is an important indicator of the use of nanomaterials in biomedicine. To study the safety of the prepared PHMB@Au NPs, blood routine and blood biochemical analysis were observed on day 0, day 1, and day 7 after subcutaneous injection of PHMB@Au NPs (9 µg mL^−1^). As shown in **Figure** **10**A–D, the results of blood biochemical analysis showed that the main blood parameters such as alanine transferase (ALT), aspartate transferase (AST), alkaline phosphatase (ALP), and blood urea nitrogen (BUN) in the control group were not significantly different from those in the experimental group (day 1 and day 7), which indicates that PHMB@Au NPs affects the liver of mice. The results of blood routine examination, including white blood cells (WBCs), red blood cells (RBCs), hemoglobin (HGB), hematocrit (HCT), mean corpuscular volume (MCV), mean corpuscular hemoglobin (MCH), platelet count (PLT), and mean platelet volume (MPV) showed that there was no significant difference between the experimental group (day 1 and day 7) and the control group, which indicated that PHMB@Au NPs showed good blood compatibility (Figure 10E–L). Meanwhile, the histological analysis of the main organs of mice using H&E staining was used to further evaluate the toxicity of PHMB@Au NPs to mice with NIR irradiation. Compared with healthy tissue without any treatment, histological analysis showed that PHMB@Au NPs showed no change in the infiltration of inflammatory cells in other organs of mice (Figures S9 and S10, Supporting Information). Therefore, the histological analysis also proved that PHMB@Au NPs have almost no biological toxicity to healthy tissues in vivo.

## 3 Conclusion

In summary, the formation of the biofilm of *S. aureus* contributes to a series of infectious diseases, bringing challenges to clinical therapeutics. The PHMB@Au NPs self‐delivery system is formed through the self‐assembly of PHMB and Au NPs, which can promote adhesion with bacteria, and quickly aggregate on the surface of *S. aureus* but not accumulate abnormally in healthy tissues. The agminated PHMB@Au NPs improved the conversion efficiency of photo‐thermal by increased NIR absorbance, resulting in thermal‐killing bacteria in vitro. Meanwhile, PHMB exhibited damage to the membrane of bacteria, which can promote PHMB@Au NPs uptake and adhere by *S. aureus* with passive targeting. Even more exciting, PHMB@Au NPs can quickly remove bacteria from wounds and subcutaneous infections in vivo, and promote wound healing without damaging the healthy tissues via PTT. The functionalized Au NPs as we are demonstrated, including antibiotics free, have potential research value in the therapy of infectious disease. Therefore, the developed PHMB@Au NPs self‐delivery system in this work may provide new materials or strategies for the field of medical anti‐infection.

## 4 Experimental Section

### Materials and Apparatus

Gold chloride trihydrate (HAuCl_4_ 3H_2_O, 48%) and sodium citrate (Na_3_C_6_H_5_O_7_ 2H_2_O, 98%) were obtained from Sigma‐Aldrich. PHMB and nitric acid (HNO_3_, 70%) were obtained from Aladdin Reagent. Reagents for cell culture including fetal bovine serum (FBS), Trypsin‐EDTA (0.25%), and Dulbecco's modified Eagle medium (DMEM) were purchased from Gibco Life Technologies. The water used in all experiments was purified by the Millipore system. The glassware used in the experiment needs to be thoroughly cleaned by soaking in 20% nitric acid and rinsing with pure water. The morphology, zeta potential, and size of Au NPs and PHMB@Au NPs were analyzed with TEM (Thermo Fisher Scientific, Talos F200S) and dynamic light scattering (Malvern, Zetasizer Nano ZS ZEN3600), respectively. UV–vis‐NIR spectroscopy of the materials was recorded on a USA CARY 5000 spectrophotometer (Agilent Technologies).

### Preparation of PHMB@Au NPs

The Au NPs were prepared according to the reference reports.^[^ ^15^ ^]^ Before the experiment, all glassware was soaked in 20% nitric acid overnight, washed with ultrapure water, and dried. Placed 50 mL of freshly prepared chloroauric acid (0.01%) solution in a 100 mL round bottom flask, heated at 60 °C and stir for 5 min, then added 4 mL of sodium citrate with a mass fraction of 1%, and continued to stir and react for 10 min until the color of the solution was not changed. Stopped heating and stirred to cool to room temperature. Separated the excess metal ions and sodium citrate from the sample and stored it in a 4 °C refrigerator. In the meantime, 10 mL PHMB (10 mg mL^−1^) was added to 10 mL of the above Au NPs by dripping and the mixture was slightly stirred for 2 h to ensure PHMB contacted the Au NPs vastly. Centrifuge and wash the above‐mentioned nano‐system many times, and finally disperse target nano‐system (PHMB@Au NPs, 1 mg mL^−1^) in the water system.

### Photothermal Effect of PHMB@Au NPs

Their temperature changes were detected at room temperature with an 808 nm laser (2 W cm^−2^, 5 min, 100 µL) and an infrared camera (E4, FLIR, USA). The heating‐cooling curves of PHMB@Au NPs (9.0 µg mL^−1^) were obtained.

Calculate the photothermal conversion efficiency (*η*) by the following formula:

(1) $$\eta \;=\frac{hS\left(\mathrm{Tmax}\;-\;\text{Tsurr}\right)\;-\;Q_{0}\;}{I\left(1\;-\;10^{-A_{808}}\right)}\;$$

(2) $$\tau_{s}=\frac{m_{d}C_{d}}{hS}$$

(3) $$Q_{0}\;=\;\text{hS}\left(\mathrm{Tmax},\mathrm{water}-\mathrm{Tsurr}\right)\;$$

The value of *τ*_s_ can be calculated from the linear regression curve in the cooling curve, the characteristic thermal time constant. m_d_ and *C*_d_ represent the mass and heat capacity of the solution, respectively. Therefore, the value of hS can be obtained. *Q*_0_ represents the background energy input in the absence of PHMB@Au NPs, and was calculated by Equation (3). where T_max_, water, and T_surr_ represent the steady‐state maximum temperature of the water, ambient room temperature, respectively. T_max_ represents the stable maximum temperature of the PHMB@Au NPs solution, and I and A_808_ represent the laser power and absorbance of PHMB@Au NPs at 808 nm, respectively. Finally, the photothermal conversion efficiency was calculated according to Equation (1).

### Cytotoxicity Study

The cytotoxicity of the materials was executed with MTT assay, and the fibroblast cells (L929) were used as the research object and purchased from the American Type Culture Collection (ATCC).^[^ ^16^ ^]^ L929 cells were cultured in DEME medium at 37 °C and 5% CO_2_, and additional 10% fetal bovine serum (FBS), penicillin (100 units mL^−1^), and streptomycin (100 mg mL^−1^) were added to the medium. Incubated PHMB@Au NPs and L929 cells together for 24 h. Simultaneously irradiated with variable‐power NIR irradiation (0.5, 1.0 and 2.0 W cm^−2^) for 5 min. The samples treated with PBS buffer were used as the control group. After 48 h of incubation, the MTT assay was used for subsequent toxicity evaluation.

### Bactericidal Effect In Vitro

*Staphylococcus aureus* (Newman strains) was used as the research object to estimate the bactericidal effect of PHMB@Au NPs in vitro.^[^ ^17^ ^]^ First, mixed of 50 µL bacterial suspension (OD_600_ 0.05) and 50 µL PHMB@Au NPs (9.0 µg mL^−1^) or 50 µL PBS was added to each well of a 96‐well plate. After incubating the above‐mixed system at 37 °C for 30 min, irradiating with 808 nm laser (2.0 W cm^−2^, 100 µL) for 5 min, and incubating for 2 h. Diluted the above‐mentioned mixed system by 625 times to count the plated colonies.

### Morphological Study

The morphological analysis of bacteria was used to record the morphological integrity changes of different materials before and after NIR irradiation treatment by scanning an electron microscope.^[^ ^18^ ^]^ The bacteria after different treatments were fixed with 2% glutaraldehyde at 4 °C for 4 h and then dehydrated with ethanol solution (50%, 70%, 90%, 95%, and 100%) gradient for 15 min, and finally spread the bacteria evenly on the silicon wafer superior. Blow‐dried gently with nitrogen, and sprayed gold on the bacterial sample on the silicon wafer by a sputtering method using SEM (Hitachi Su 8010) instrument at 3.0 kV.

### Biofilm Assay of S. Aureus

*S. aureus* was cultured in a 6‐well microtiter plate (Corning, NY, USA) to form a static biofilm. For the biofilm inhibition assay, approximately 3 mL of overnight bacterial culture was diluted in fresh tryptic soy broth (TSB) medium to a final OD600 value of 0.05 and transferred to a 6‐well plate at 9 µg mL^−1^ PHMB@Au NPs. Incubated the plate for a further 48 h at 37 °C. The biofilm of *S. aureus* was stained with CV for quantification. In short, the weakly adherent planktonic cells were removed from the 6‐well microtiter plate by washing three times with PBS buffer. Dried the adhered biofilm and stained with 0.1% (w/v) CV for 15 min. Removed excess dye by washing the plate 3 times with PBS. The CV was dissolved from the stained biofilm by adding 30% acetic acid, and the biofilm was quantified by measuring the absorbance at 595 nm. Each experiment was performed in triplicate.

### Hemolysis Assay of S. Aureus

The assay of PHMB@Au NPs inhibiting hemolysis activity of *S. aureus* was carried out according to previous literature reports. The rabbit red blood cell hemolysis test was used to detect the hemolysin activity secreted by *S. aureus*. *S. aureus* was incubated in 9 µg mL^−1^ PHMB@Au NPs until the OD600 value reached 1.0. The bacterial supernatant medium was collected by centrifugation. Fresh rabbit red blood cells were washed 3 times with PBS buffer and diluted 50 times with TSB medium. Thereafter, 0.5 mL of the prepared red blood cell solution was mixed with 0.5 mL of bacterial culture medium. At the same time, the TSB medium served as a control. Incubated the mixture for another 30 min at 37 °C. The supernatant of the mixture was collected by centrifugation at 10 000 ×g for 10 min. The hemoglobin released in the supernatant was determined by measuring the absorbance at 595 nm. Use the following formula to calculate the red blood cell lysis percentage: [(SN)/(PN)]×100%, where PN is the OD595 value of the negative control TSB medium, and SN is the OD595 value of the analyzed sample. Three determinations were performed independently and each determination was measured in triplicate.

### Bactericidal Effect In Vivo

To further explore the bactericidal effect in vivo, the subcutaneous abscess model and wound healing model were developed in BALB/c mice and applied with PHMB@Au NPs (9 µg mL^−1^) injection combined with NIR irradiation.^[^ ^19^ ^]^ For the subcutaneous abscess model, the mice were anesthetized and injected subcutaneously with *S. aureus* (50 µL, 1.0 × 10^8^ CFU mL^−1^) on their backs, and the model was confirmed successfully after 24 h. At this time, an obvious abscess area will be seen subcutaneously in BALB/c mice, indicating that the subcutaneous abscess model was successfully established. For the wound healing model, first, remove the back hair and cut out a 6.0 mm diameter wound on the back with scissors. The wound site was infected with *S. aureus* suspension (20 µL, 1.0 × 10^8^ CFU mL^−1^) to establish a mouse model of *S. aureus* wound infection. During the photothermal antimicrobial therapy, the NIR image was used to record the temperature changes around the abscess and wound to evaluate the in situ imaging performance of PHMB@Au NPs. The wound area of BALB/c mice was calculated by Image J software. The standard plate counting method was used to quantitatively evaluate the bactericidal effect of different groups of mice. Hematoxylin and eosin (H&E) staining methods were used for histological analysis of skin sections to evaluate the safety assessment of PHMB@Au NPs during photothermal antimicrobial therapy under NIR irradiation. All test groups and control groups contained five parallel groups for repeatability evaluation.

### Histological and Immunofluorescence Analysis

After the 7th day, the wound tissues of the mice were collected, fixed with 4% paraformaldehyde, and embedded in paraffin to slice into 5 µm thick tissue sections. For histological analysis, sections were stained with hematoxylin and eosin, Gram and Masson trichrome stains after deparaffinization and rehydration. For immunofluorescence staining, deparaffinized and rehydrated sections were heat‐induced antigen retrieval in citrate buffer (10 mm, pH 6.0) at 98 °C for 10 min. After infiltration, 10% goat serum was used to block non‐specific binding for 1 h and 10 min. To assess the level of angiogenesis, rabbit anti‐CD31 primary antibody was used and then incubated with goat anti‐rabbit secondary antibody. Used TNF‐*α*, CD86, TGF‐*β*, and CD206 primary antibodies and corresponding secondary antibodies to determine the phenotype of macrophages in wound tissue. The nuclei were counterstained with DAPI for 10 min. A fluorescence microscope (Olympus, Japan) was used to acquire immunofluorescence images, and ImageJ software was used for quantification.

### Statistical Analysis

The data of experiments including anti‐biofilm, hemolysin inhibition, antimicrobial activity in vitro and in vivo, and immunofluorescence analysis were expressed as mean values ± standard deviation (SD). The above experimental data was repeated three times in parallel (n = 3), and the data was counted by a two‐tailed Student's t‐test, and its statistical value was expressed in the following proportion: **P <0.01 and ***P <0.001. Graph analysis was performed using GraphPad Prism 8.0 (GraphPad Software, USA).

### Live Subject Statement

All animal procedures were performed under the Guidelines for Care and Use of Laboratory Animals of Wenzhou Medical University and approved by the Animal Ethics Committee of Wenzhou Medical University (No. SYXK2021‐0020).

## Conflict of Interest

The authors declare no conflict of interest.

## Supporting information

Supporting Information

## Data Availability

Research data are not shared.
