# Supplementary material for: A Vehicle‐Free Antimicrobial Polymer Hybrid Gold Nanoparticle as Synergistically Therapeutic Platforms for Staphylococcus aureus Infected Wound Healing
Source: Adv Sci (Weinh). 2022 Mar 10;9(14):2105223. doi: 10.1002/advs.202105223 (PMC9108595; doi:10.1002/advs.202105223)
Supplement: Supplementary file 1 — Supporting Information [file ADVS-9-2105223-s001.pdf]

ADVANCED SCIENCE Open Access

Supporting Information

for *Adv. Sci.*, DOI 10.1002/advs.202105223

A Vehicle-Free Antimicrobial Polymer Hybrid Gold Nanoparticle as Synergistically Therapeutic Platforms for *Staphylococcus aureus* Infected Wound Healing

Xiaojun He, Lixiong Dai, Lisong Ye, Xiaoshuai Sun, Obeng Enoch, Rongdang Hu, Xingjie Zan, Feng Lin* and Jianliang Shen**

Supporting information

A Vehicle-free Antimicrobial Polymer Hybrid Gold Nanoparticle as Synergistically Therapeutic Platforms for *Staphylococcus aureus* Infected Wound Healing

Xiaojun He,[1,§] *Lixiong Dai,*[2,§] *Lisong Ye,*[4,§] *Xiaoshuai Sun,*[4] *Obeng Enoch,*[1] *Rongdang Hu,*[4] *Xingjie Zan,*[1,2,3*] *Feng Lin,*[5*] and *Jianliang Shen*[1,2,3*]

[1]School of Ophthalmology & Optometry, School of Biomedical Engineering, Wenzhou Medical University, Wenzhou, Zhejiang 325035, China

[2]Wenzhou Institute, University of Chinese Academy of Sciences, Wenzhou, 325000, China

[3]Oujiang Laboratory (Zhejiang Lab for Regenerative Medicine, Vision and Brain Health), Wenzhou, Zhejiang 325001, China

[4]School of Stomatology, Wenzhou Medical University, Wenzhou, Zhejiang 325035, China

[5]Department of gynecology, the First Affiliated Hospital of Wenzhou Medical University, Wenzhou 325000, China

All authors have approved the final version of this manuscript.

[§]These authors contributed equally to this work.

*Address correspondence: xjzan2000@hotmail.com (X. Zan), lin801026@163.com (F. Lin), shenjl@ucas.ac.cn (J. Shen)

Figure/Caption:

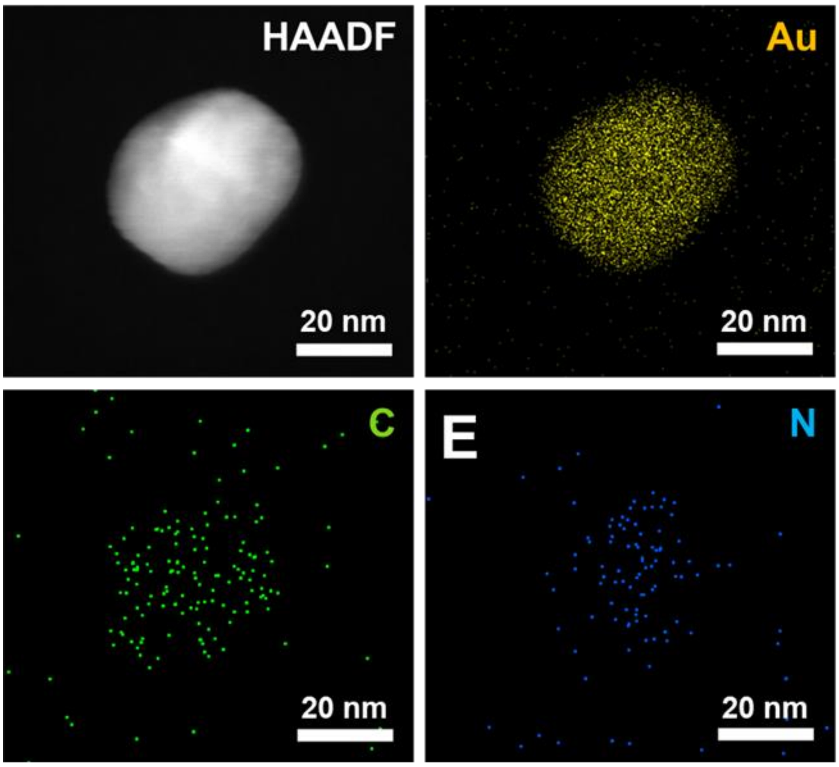

Figure S1. STEM image of PHMB@Au NPs and elemental mappings of Au, C, and N. Scale bars=20 nm.

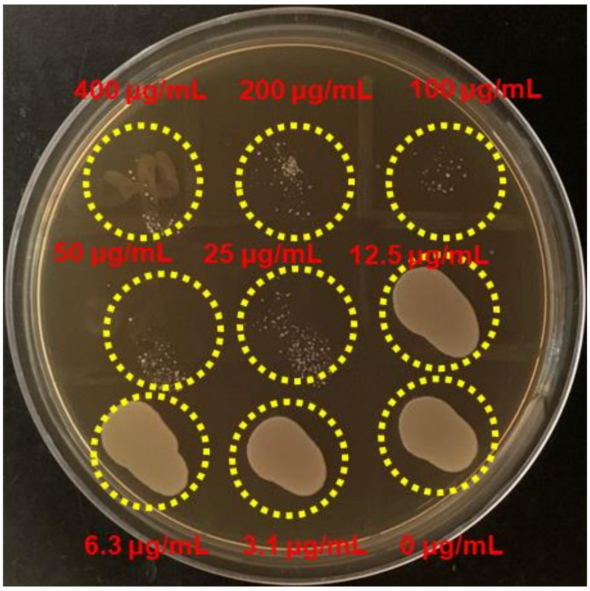

Figure S2. Photographic images of the colonies of *S. aureus* treated by various concentrations of polyhexamethylene biguanide.

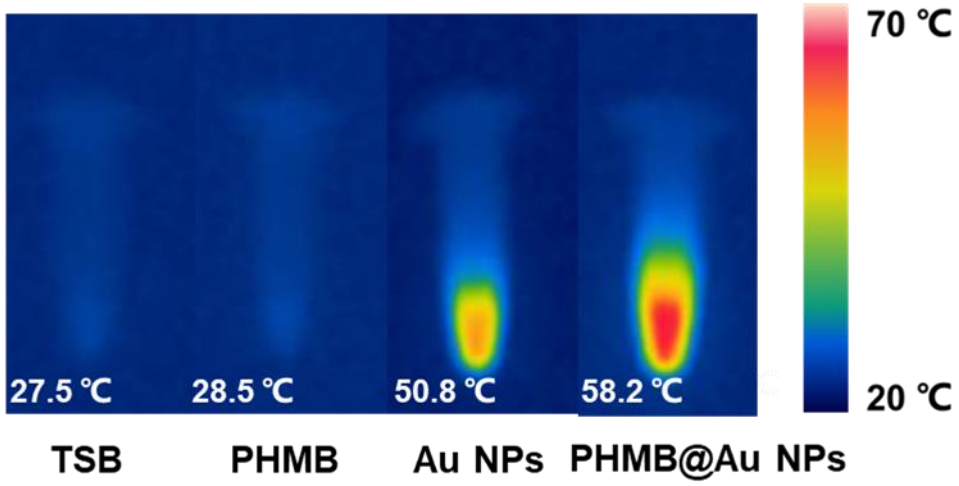

Figure S3. Thermographic images of trypticase soy broth (TSB), PHMB, Au NPs, and PHMB@Au NPs in water solution under 808 nm irradiation (2.0 W cm^{-2}).

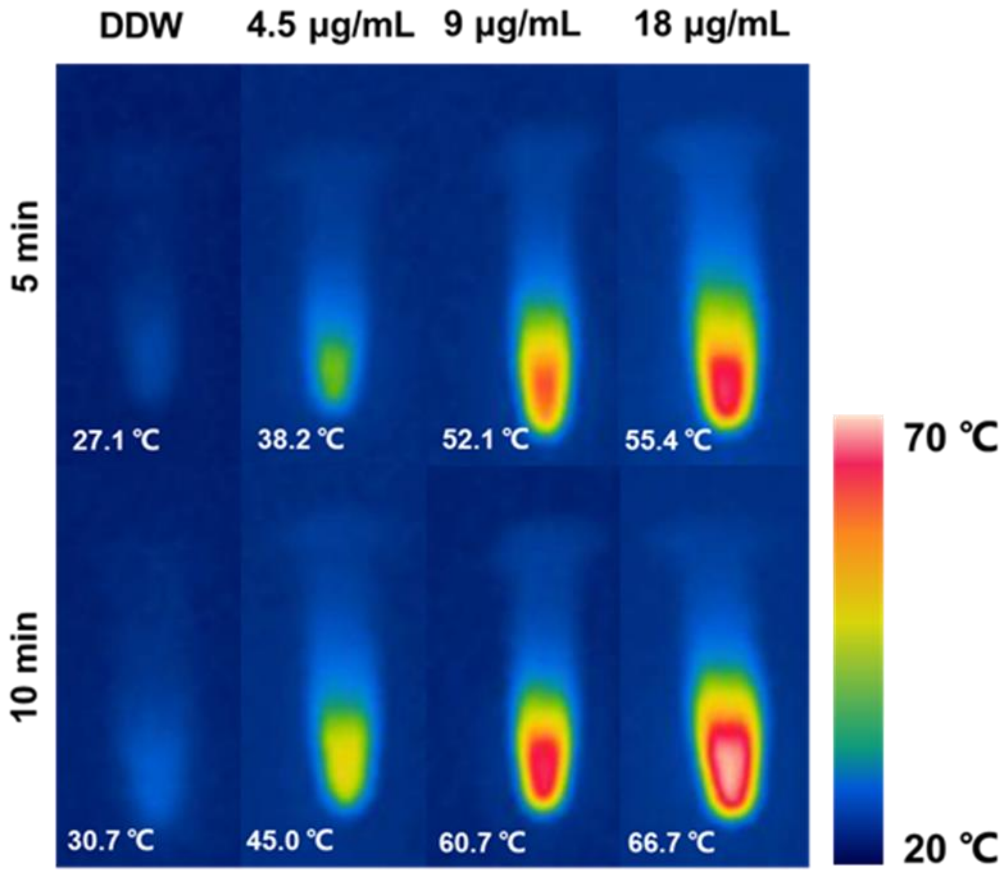

Figure S4. Thermographic images of PHMB@Au NPs with various concentrations (0, 4.5, 9.0, and 18 μg/mL) in water solution under 808 nm irradiation (2.0 W cm^{-2}).

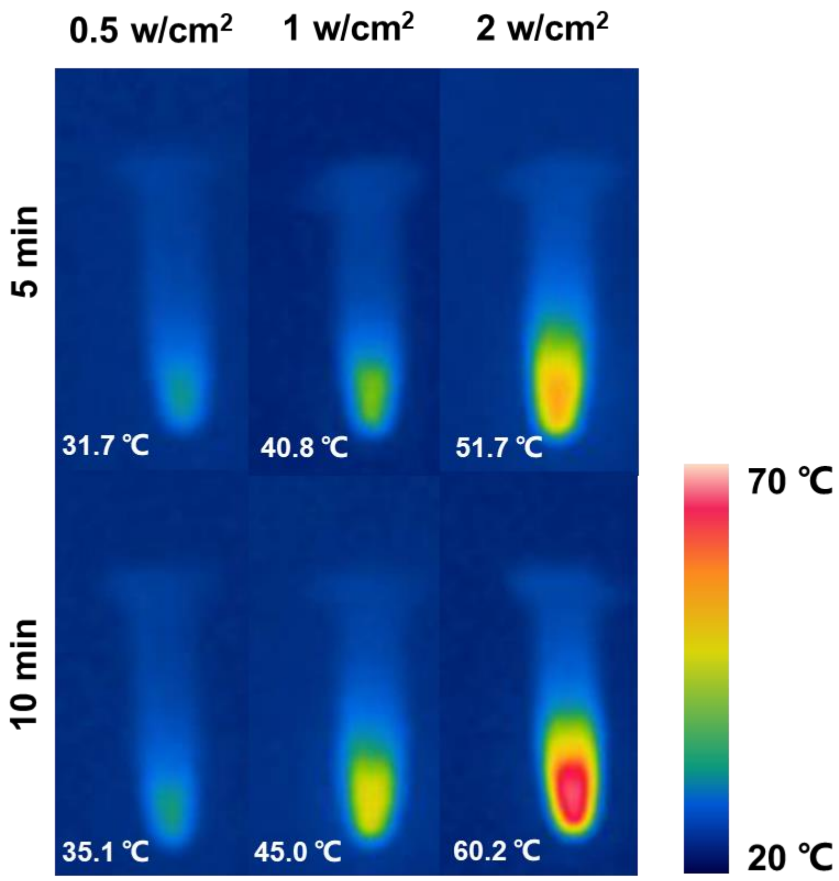

Figure S5. Thermographic images of PHMB@Au NPs (9.0 μg/mL) in water solution under with different power 808 nm irradiation.

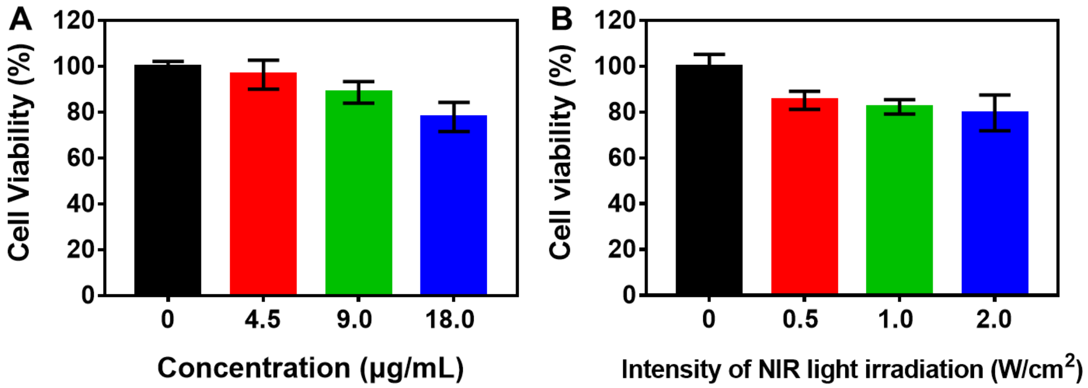

Figure S6. (A) Cell viability of L929 cells after treated with different concentration of PHMB@Au NPs (0, 4.5, 9.0 18.0 μg/mL). (B) Cell viability of L929 cells after treated with PHMB@Au NPs under different intensity of NIR irradiation for 5 min.

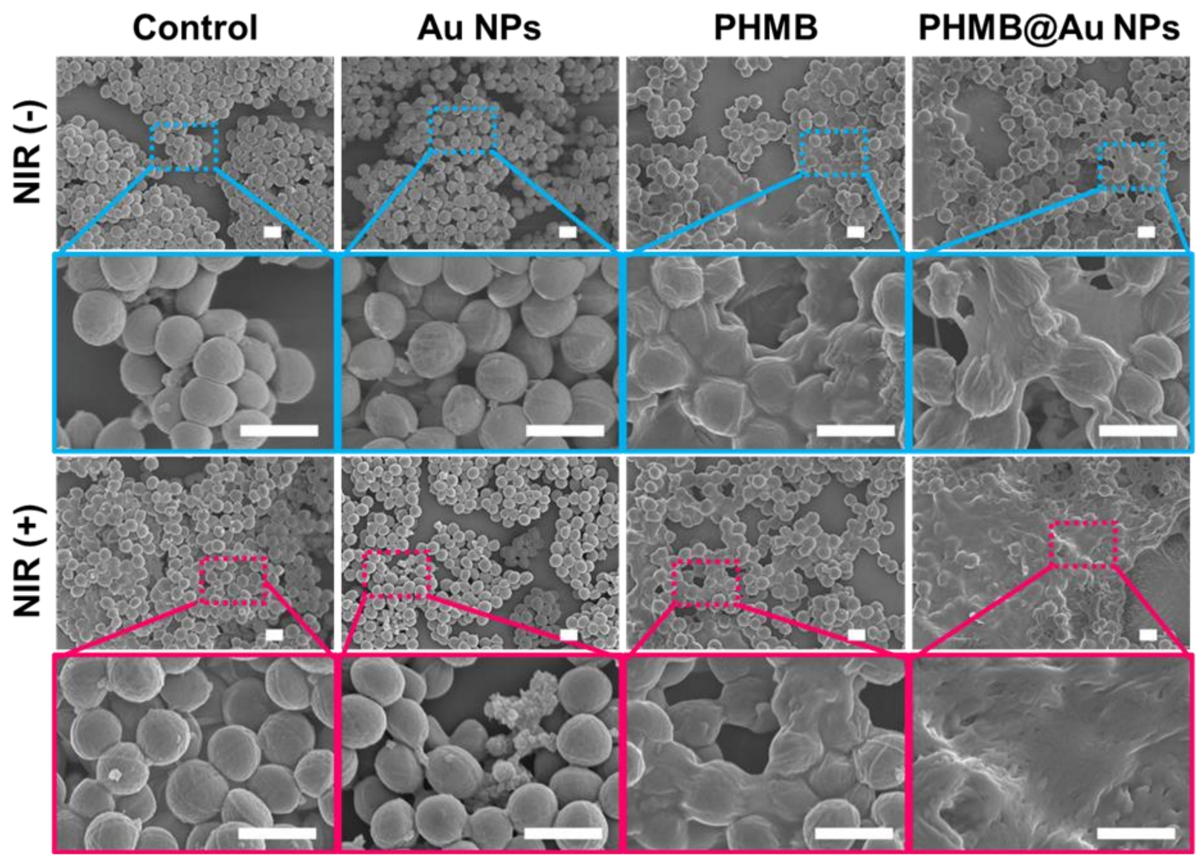

Figure S7. SEM images (including local zoom) of *S. aureus* after 5 min of 808 nm laser irradiation (2.0 W cm^{-2}) with the treatment of PHMB@Au NPs (9.0 μg/mL). The control groups were imaged in the absence and presence of irradiation treatment with a TSB medium. scale bar: 1.0 μm.

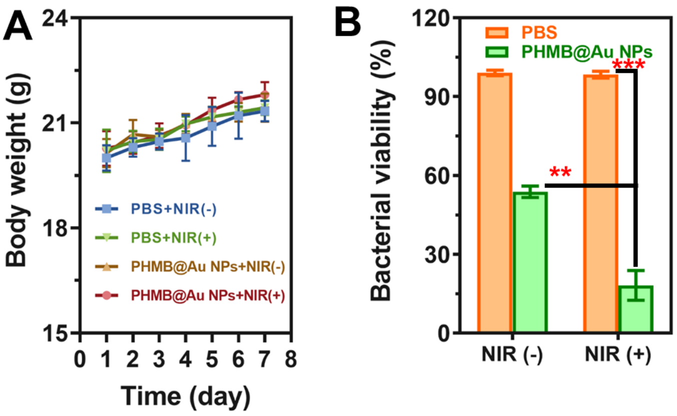

Figure S8. (A) Time-dependent body-weight curves of mice after different treatments. (B) Quantitative statistics of the number of bacterial colonies through standard plate counting assay (n=3). **P <0.01 and ***P <0.001.

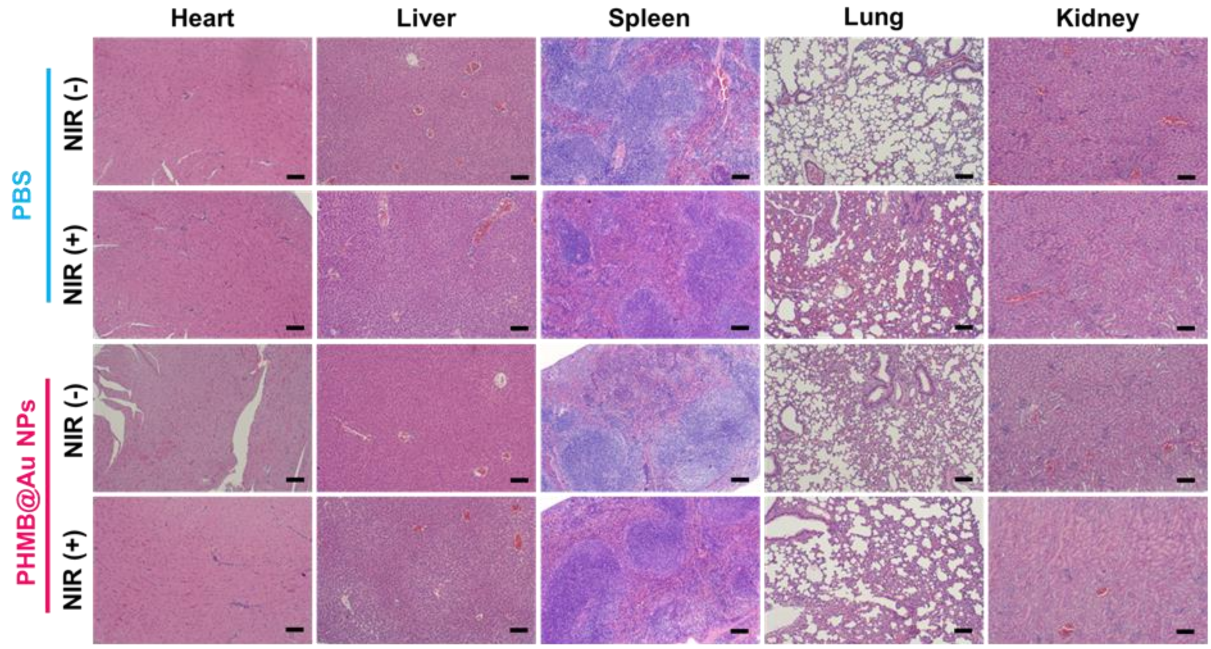

Figure S9. Hematoxylin and Eosin stain (H&E) of major organs tissue sections of infected mice with different treatments in subcutaneous abscess model (Day 7). Scale bar, 200 μm.

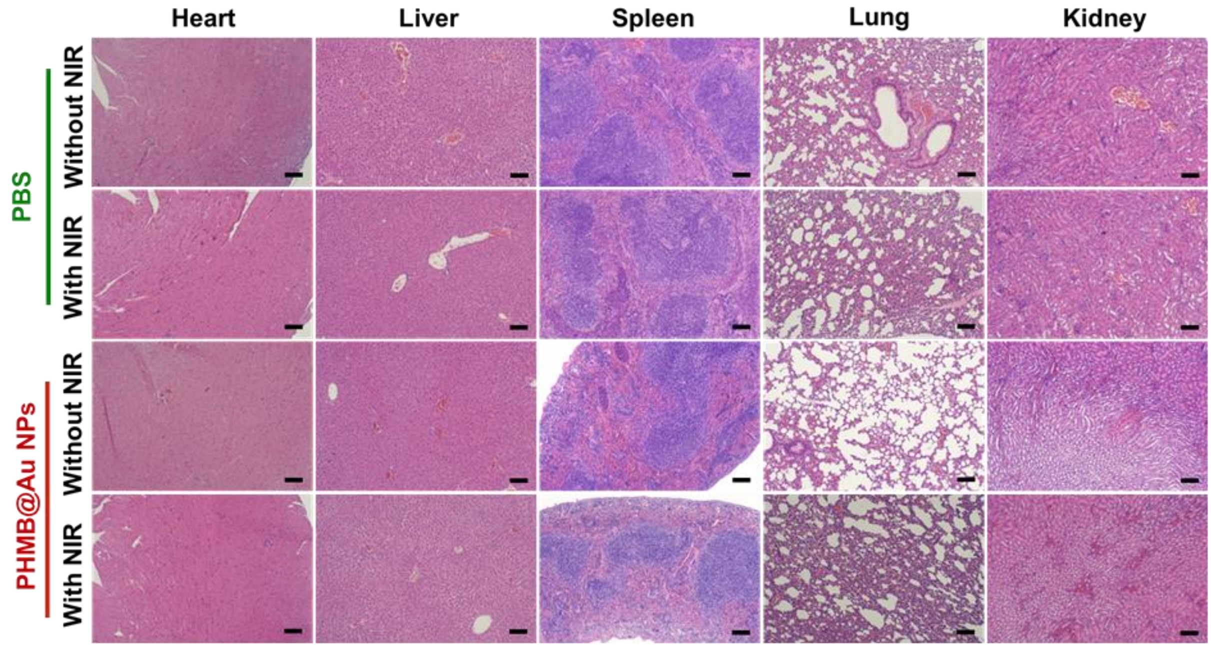

Figure S10. Hematoxylin and Eosin stain (H&E) of major organs tissue sections of infected mice with different treatments in wound healing model (Day 7). Scale bar, 200 μm.
